# Understanding Pediatric Experiences With Symptomatic Varicoceles: Mixed Methods Study of an Online Varicocele Community

**DOI:** 10.2196/50141

**Published:** 2024-10-10

**Authors:** Grace E Sollender, Tommy Jiang, Ilana Finkelshtein, Vadim Osadchiy, Michael H Zheng, Jesse N Mills, Jennifer S Singer, Sriram V Eleswarapu

**Affiliations:** 1 Department of Urology University of California, Los Angeles Los Angeles, CA United States; 2 Department of Urology Stanford University Palo Alto, CA United States

**Keywords:** adolescents, online support, online forums, peer support, natural language processing

## Abstract

**Background:**

Varicoceles affect up to 30% of postpubertal adolescent males. Studying this population remains difficult due to this topic’s sensitive nature. Using the popularity of social media in this cohort and natural language processing (NLP) techniques, our aim was to identify perceptions of adolescent males on an internet varicocele forum to inform how physicians may better evaluate and counsel this pediatric population.

**Objective:**

We aimed to characterize themes of discussion and specific concerns expressed by adolescents using a mixed methods approach involving quantitative NLP and qualitative annotation of an online varicocele community.

**Methods:**

We extracted posts from the Reddit community “r/varicocele” (5100 members) with criteria of discussant age ≤21 years and word count >20. We used qualitative thematic analysis and the validated constant comparative method, as well as an NLP technique called the meaning extraction method with principal component analysis (MEM/PCA), to identify discussion themes. Two investigators independently interrogated 150 randomly selected posts to further characterize content based on NLP-identified themes and calculated the Kaiser-Meyer-Olkin (KMO) statistic and the Bartlett test. Both quantitative and qualitative approaches were then compared to identify key themes of discussion.

**Results:**

A total of 1103 posts met eligibility criteria from July 2015 to June 2022. Among the 150 randomly selected posts, MEM/PCA and qualitative thematic analysis separately revealed key themes: an overview of varicocele (40/150, 27%), management (29/150, 19%), postprocedural experience (28/150, 19%), seeking community (26/150, 17%) and second opinions after visiting a physician (27/150, 18%). Quantitative analysis also identified “hypogonadism” and “semen analysis” as concerns when discussing their condition. The KMO statistic was >0.60 and the Bartlett test was <0.01, indicating the appropriateness of MEM/PCA. The mean age was 17.5 (SD 2.2; range 14-21) years, and there were trends toward higher-grade (40/45, 89% had a grade of ≥2) and left-sided varicoceles. Urologists were the topic of over 50% (53/82) of discussions among discussants, and varicocelectomy remained the intervention receiving the most interest. A total of 60% (90/150) of discussants described symptomatic varicoceles, with 62 of 90 reporting pain, 24 of 90 reporting hypogonadism symptoms, and 45 of 90 reporting aesthetics as the primary concern.

**Conclusions:**

We applied a mixed methods approach to identify uncensored concerns of adolescents with varicoceles. Both qualitative and quantitative approaches identified that adolescents often turned to social media as an adjunct to doctors’ visits and to seek peer support. This population prioritized symptom control, with an emphasis on pain, aesthetics, sexual function, and hypogonadism. These data highlight how each adolescent may approach varicoceles uniquely, informing urologists how to better interface with this pediatric population. Additionally, these data may highlight the key drivers of decision-making when electing for procedural management of varicoceles.

## Introduction

Varicoceles affect 5% to 30% of postpubertal male adolescents aged 12 to 18 years [1]. Definitive management guidelines for this population remain poorly defined, but generally speaking, varicocele repair is recommended to preserve or improve fertility in pain-free adolescents with ipsilateral reduction in testicular size or abnormal semen analysis [2,3]. Families are presented with a range of management options, including longitudinal observation with serial follow-up versus an interventional approach. However, there remains a paucity of evidence that procedural treatment of adolescent varicocele is associated with improved testicular size or sperm concentration, and ultimate effects on fertility and paternity rates are unknown [3-5]. Because of the uncertain natural history, the treatment algorithm is complex to navigate for health care providers and often relies on patient preferences.

Social media provides a unique opportunity to understand adolescents’ viewpoints and anxieties on how they navigate varicocele diagnosis, management, and treatment. Reddit is one such discussion platform; it has over 430 million monthly active users globally and has gained popularity in the discussion of health-related topics [6]. “r/varicocele” is the largest varicocele discussion board on Reddit and provides a platform for users to anonymously share their experiences, seek knowledge, disseminate resources, provide advice, belong to a community, and interact with each other [7]. Analysis of these sites can be informative for urologists to understand some concerns among adolescents with varicoceles and may aid in understanding key drivers of decision-making. Additionally, the emergence of natural language processing (NLP) techniques has led to innovative uses in health care; however, its use to examine patient-centered experiences is limited. There remains discussion on how best to approach these methodologies [8]. Previous work has highlighted the role of web-based forums as a means of eliciting the sexual health problems of a group that has been reluctant to consult practitioners, peers, and others for personal health advice and information [9,10]. Additionally, recent studies have leveraged these discussions to uncover patient perspectives on erectile dysfunction, hypogonadism, and nephrolithiasis [11-13]; however, use of social media specific to an adolescent varicocele population has not been studied.

Due to the sensitive nature of the topic, there remains limited literature evaluating perceptions of varicoceles among the pediatric population. Using a mixed methods approach, our aim was to understand adolescent patient perspectives and concerns on varicocele management. We hypothesized that the content of online discussions posted by adolescents with varicoceles can be classified into themes that may inform how physicians evaluate, counsel, and treat this patient population. Specifically, we predicted discussants would focus their chief concerns on sexual function, aesthetics, hypogonadism, and pain. This study may help health care providers improve interactions and communications with adolescent patients and ultimately allow for the provision of patient-centered care [10].

## Methods

### Overview

We used a convergent design for this mixed methods research study, whereby qualitative and quantitative data were collected and analyzed during a similar timeframe [14]. Prior studies have used this methodology to answer similar research questions and highlight the complementary role of convergent design [11,12,15]. The study comprised four phases: (1) data extraction from Reddit, (2) classic qualitative thematic analysis, (3) NLP-based quantitative analysis to identify themes of discussion, and (4) manual mining of a subset of posts. We defined a post as the discussant’s original text entry onto the forum.

To obtain these data, we retrospectively extracted posts from the largest varicocele discussion board on the Reddit community, r/varicocele [7]. A word count criterion (>20 words per post) was then used to exclude potential spam, deleted texts, and posts containing only a link to a website. All posts were then manually evaluated for self-reported age ≤21 years. All remaining posts underwent NLP-based quantitative analysis, while only a subset underwent qualitative thematic analysis and manual mining.

Demographic information was manually solicited from the cohort of posts since authors frequently started discussions by providing information about their age and background. One challenge for data acquisition was the free-form nature inherent to the website; thus, all information was self-reported and not verified. In the event of ambiguity, we did not collect demographic data from that post. Therefore, all analyzed posts had an identifiable, self-reported age ≤21 years. For other variables such as varicocele laterality or grade, this information was not uniformly available; however, a large subset self-reported this data. We extracted the following data: demographics (age, sex), relevant medical history (varicocele grade, laterality), health care visits (emergency room, primary care physician, urologist, interventional radiology, endocrinologist), treatment modalities discussed (observation, embolization, varicocelectomy), and any specific testing that may have occurred (such as bloodwork or semen analysis and imaging, such as ultrasound). Discussions that mentioned multiple modalities were categorized separately and therefore not doubly counted.

### Qualitative Thematic Analysis

We performed qualitative thematic analysis using 150 randomly selected posts. This number was selected based on prior studies demonstrating this quantity of posts was required to achieve thematic saturation, whereby no additional themes were identified with analysis of each additional post [12,13,15]. The study protocol specifically allowed for the addition of 50 more posts if thematic saturation was not reached by 150. This process would have been iteratively repeated until thematic saturation was reached. The basis of our analysis was validated grounded theory and a constant comparative methodology [16,17]. Two investigators independently analyzed text from each post to identify preliminary themes. These preliminary themes were then discussed among all authors before finalizing summary themes. Summary themes were independently identified for the 150 selected posts and agreed upon by all reviewers. This method has been previously validated [13].

### NLP-Based Quantitative Analysis

Posts were separately subjected to an NLP technique called the meaning extraction method (MEM) with principal component analysis (PCA). MEM/PCA tracks words that cluster together to derive themes quantitatively [18]. This approach has been previously validated to reveal information about individuals’ personalities, communication strategies, and behaviors [18-20].

To automate MEM, we used the topic modeling application Meaning Extraction Helper (MEH; version 2; Meaning Extraction Project). Using MEH, each post was deconstructed into its component words, while articles, prepositions, and transition words were removed. Remaining words were ranked by their frequencies of appearance in each post. Words were then subjected to PCA with varimax rotation using SPSS (version 25; IBM Corp). PCA identified clusters of words that frequently appeared together. Each word was conferred a factor loading, representing the correlation coefficient between the word and the cluster to which it belonged. We assigned a descriptive theme to each cluster based on the words within it. To assess the applicability of PCA to each dataset, we calculated the Kaiser-Meyer-Olkin (KMO) statistic, which is a measure of sampling adequacy (>0.60), as well as the Bartlett test for sphericity, which determines whether significant correlations exist among variables of interest [21].

### Ethical Considerations

All data used were publicly available and only used to observe public behaviors of patients experiencing symptomatic varicoceles. For further protection, all data obtained were anonymous, further deidentified during data analysis, and only available to pertinent research staff throughout the study. The study was exempt from institutional review board evaluation due to its use of anonymized, publicly available data from the internet.

## Results

### Overview

We extracted 8542 posts from r/varicocele, of which 6804 met the inclusion criterion of being posted from inception in July 2015 to June 2022. A total of 1103 posts were identifiably posted by a discussant with a self-reported age ≤21 years. All 1103 posts underwent quantitative NLP analysis, while 150 randomly selected posts underwent qualitative analysis and manual annotation. Multimedia Appendix 1, Table S1, contains additional representative quotations that feature each theme described in the qualitative analysis. Of note, representative quotations have been abridged in the interest of space, and profane language has been filtered.

Demographics are presented in Table 1. Patients who reported specifics of their condition were predominantly adolescent and male with a left-sided varicocele that trended toward a higher grade in presentation.

**Table 1:** Demographic details of the 150 randomly selected patients that underwent qualitative analysis. Data were self-reported on the social media platform Reddit (r/varicocele) from July 2022 until June 2022. Posts were eligible if they self-reported age; however, other reported details were not universally disclosed.

Demographics		Values
Age (years), mean (SD; range)		17.5 (2.2; 14-21)
**Varicocele grade (patients), n**		
	Not mentioned	105
	Grade 1	5 (+1^a^)
	Grade 2	10 (+3^a^)
	Grade 3	30 (+2^a^)
**Sidedness of varicocele (patients), n**		
	Not mentioned	102
	Left	76
	Right	0
	Bilateral	8
**Timeline of post (patients), n**		
	Prior to procedure	120
	After procedure	30
**Poster (patients), n**		
	Individual	146
	Other	4

^a^Posts by some patients with bilateral varicoceles mentioned the grade of each testicle.

### Qualitative Thematic Analysis

Young men turned to social media to receive education about varicoceles. It was common for discussants to ask if varicoceles were associated with multiple conditions, including hypogonadism, infertility, cessation of puberty, chronic testicular pain, and malignancy.

I’m a 17 year old [sic] male and I just had lab work done to determine my testosterone levels since I’ve been experiencing ED, fatigue, depression, and such.... What do you guys think? Is there a definitive answer on varicoceles and T?

Discussants sought additional information about the efficacy of supplements, which included horse chestnut, diosmin plus, and L-carnitine/acetyl-L-carnitine.

One of my friends also has a grade 3 varicocele and told me about how he takes horse chestnut extract pills.

Discussants at all stages of management used social media to find shared experiences among a common community. Young men often asked if people in the community had experienced similar symptomology.

I can still feel it but it’s not as sensitive as the right one which I find to be concerning. Does anyone have a similar experience to this? Also, after having read through postings, y’all seem like a super supportive bunch and it makes me happy to see I’m not alone.

Many patients were distressed by their varicoceles and used r/varicocele as a source of emotional support. A subset of discussants felt their varicocele made them less masculine, and many discussants attributed poor sexual performance to varicocele presence.

It really started to stress me when it flared up and hindered my sex life. What really gets me angry is talking to doctors who say me feeling less masculine is all in my head!!!! I am deeply depressed to the point I’m contemplating suicide.

Young men used discussion forums as a substitute for doctors’ visits. It was common to ask the community to diagnose their condition instead of seeking evaluation with a health care provider.

Im [sic]16 years old and ive [sic] had pain in my left testicle/veins for 4 days now. My testicle feels heavy and i [sic] think it has gotten bigger. What is going on? Is this a varicocele?

Separately, discussants also used the forum to interpret lab/imaging results and receive a second opinion.

**Prolactin: 27.2 µg/L** (range <20)

...

**SHBG: 36 nmol/L** (18.3-54.1)

Seems Prolactin and LH are high, while FSH and Free Test are low. What could these values mean?

Discussants also queried about treatment options for their varicoceles, including conservative versus procedural management. For surgical management, discussants often worried about procedural risks and benefits.

So I just turned 20 and have had a huge grade 3 left side. The worry for me is the size of my left testicle. It’s noticeably smaller than my right and since I sag constantly, I’m worried about the damage. If I get a surgery this year, will the size return to normal? What are the downsides?

Additionally, discussants asked if they were candidates for surgical management.

Is that enough to warrant embolization? I know embolization is a pretty safe procedure but I guess the idea of coils just being kind of left in my body scare [sic] me a bit. If the pain/discomfort I get from my varicocele is livable, do I not bother?

For the subset of individuals who underwent procedural varicocele management, it was common to discuss postoperative experiences. Discussants frequently shared their experiences regarding procedural side effects, as well as concerns regarding nonimproving varicoceles following procedural management. Adolescents were concerned about postoperative aesthetics. Additionally, those who successfully recovered reviewed what remedies they found helpful to address common postoperative concerns.

Just had microsurgery. I’m just a 17y/o kid and wanted to get it out before college. I’ve had a few sexual partners mention it after the fact, and I feel embarrassed when they mention it because I don’t know how to explain, so its [sic] an insecurity as well. I want to know if your varicose veins will go away post op, or if there is anyway it’ll get smaller.

### Quantitative Thematic Analysis

Results of NLP are shown in Table 2. Using MEM/PCA, we identified thematic word clusters, which included words with a factor loading >0.30. The following themes emerged: concerns with surgery, basics of varicoceles, management, hypogonadism, varicocele evaluation, and semen analysis. A KMO result of >0.60 and a Bartlett test value <0.01 indicated the appropriateness of MEM/PCA for these datasets.

**Table 2:** Quantitative analysis results. All 1103 eligible posts from r/varicocele (July 2015 to June 2022) underwent a natural language processing technique called the meaning extraction method with principal component analysis (PCA). Using the Meaning Extraction Helper, all textual data were deconstructed into their component words. PCA then identified word clusters that frequently appeared together and assigned a factor loading (FL) score.

Topics/words		FL
**Concerns with surgery**		
	Bed	0.548
	Walk	0.492
	Room	0.470
	Hospital	0.457
	Wake	0.444
	Sit	0.435
	Swelling	0.432
	Hour	0.421
	Day	0.401
	Incision	0.400
	Move	0.389
	Recovery	0.381
	Update	0.377
	Stay	0.335
	Painful	0.334
	Leg	0.324
	Better	0.318
	Week	0.318
	Easy	0.313
	Area	0.308
	Pain	0.308
	Push	0.307
	Longer	0.304
	Wear	0.303
	Under	0.303
	Stand	0.302
	Lay	0.302
	Night	0.301
**Basics of varicoceles**		
	Young	0.464
	People	0.387
	Condition	0.375
	Research	0.346
	Mind	0.336
	Live	0.333
	Lead	0.327
	Worse	0.315
	Life	0.312
	Doctor	0.308
	Affect	0.303
	Call	0.301
	Care	0.300
**Management**		
	Procedure	0.545
	Coil	0.516
	Embolization	0.483
	Radiologist	0.465
	Perform	0.399
	Microsurgery	0.324
	Risk	0.306
	Post	0.304
**Hypogonadism**		
	Testosterone	0.538
	Libido	0.523
	Low	0.479
	Range	0.436
	Test	0.434
	Erection	0.402
	Level	0.393
	Blood	0.386
	Normal	0.384
	Sex	0.376
	Weight	0.356
	Eat	0.331
	Lift	0.327
**Varicocele evaluation**		
	Testicle	0.538
	Left	0.467
	Ultrasound	0.403
	Lump	0.369
	Right	0.366
	Dull	0.329
	Ache	0.326
	Side	0.318
	Small	0.306
**Semen analysis**		
	Count	0.560
	Sperm	0.556
	Analysis	0.554
	Semen	0.500
	Worm	0.321

### Management and Health Care Provider Discussions

The most common reason discussants sought out r/varicocele was to learn about varicoceles (40/150, 27%); however, it remained common for adolescents to seek communal experiences (26/150, 17%), ask questions about management (29/150, 19%), share postoperative experiences (28/150, 19%), and receive information to supplement a doctor’s visit (27/150, 18%).

When discussing experiences, at least 60% of discussants (90/150) reported symptomatic varicoceles. Most commonly, patients reported pain (62/90) and signs of hypogonadism (24/90). A sizable cohort of patients discussed their concern with varicocele aesthetics (45/90).

When examining specific management, discussants were most likely to mention their experiences with urologists (53 of 82 posts mentioning a health care provider) and most likely to discuss varicocelectomy when treatment was a topic of discussion (54/108, 50%) (Table 3). Additionally, 14% (21/150) mentioned an imaging modality, 52% (78/150) mentioned a physical exam finding, and 25% (38/150) mentioned testing for an associated condition (ie, hormone testing and semen analysis).

The convergent mixed methods design used in this study allowed for analysis of areas of convergence or divergence between quantitative and qualitative datasets. The results of the qualitative analysis were verified by the quantitative analysis. Specifically, the quantitative portion provided a statistical and objective backbone to identifying topics of discussion.

**Table 3:** Self-reported health care providers mentioned and interventions discussed. Note that some posts list more than one health care provider type.

			Posts, n (%)
**Health care providers mentioned (n=82)**			
	Urologists	53 (65)	
	Interventional radiologists	3 (4)	
	Primary care physician	15 (18)	
	Emergency department	1 (1)	
	Endocrinology	1 (1)	
	Multiple	9 (11)	
**Interventions discussed (n=108)**			
	Conservative	26 (24)	
	Embolization	27 (25)	
	Varicocelectomy	55 (51)	

## Discussion

### Principal Findings

This study represents an evaluation of a social media community focused exclusively on varicoceles using a mixed methodology. Both quantitative and qualitative thematic analysis revealed that a major reason adolescents engaged with internet discussion forums was to educate themselves on basic information related to varicoceles and associated symptoms, discuss postoperative expectations, and guide decision-making for interventional management. Additionally, this platform served as an outlet for a second opinion.

We identified that 26% (39/150) of discussants either described symptoms of hypogonadism or asked about serum testosterone testing. The prevalence of isolated hypogonadism in male adolescents or adults with varicoceles has not been described; however, prior research has examined the effect of surgical repair on changes in serum testosterone level in male adults who underwent varicocelectomy for infertility, finding an increase in serum testosterone levels following repair [22]. Despite this finding, the surgical treatment of varicocele for hypogonadism in male adults is controversial. Furthermore, diagnosing hypogonadism in adolescents is even less clear, since classic symptoms of hypogonadism commonly found in older male adults, such as decreased libido or erectile dysfunction, are not usually present in the younger demographic. Additionally, there is no established reference range for serum testosterone in the developing male adolescent [23]. Understanding adolescent concerns surrounding hypogonadism is important so that this population can be correctly educated on the uncertain relationship between uncorrected adolescent varicocele and concurrent hypogonadism.

At every point in management, patients discussed sexual function, aesthetics, and hypogonadism as key drivers of decision-making. A sizable cohort discussed their concern with varicocele aesthetics, commenting that partners had mentioned it in the context of sexual activity, which often made discussants feel embarrassed or insecure. Others felt it negatively affected their masculinity. Having a varicocele may negatively impair a male adolescent’s self-esteem and hamper their ability to establish a positive sexual identity, as a large portion of self-esteem during adolescence is derived from sexual identity [24]. Thus, it is important for urologists to explore the degree of concern associated with the aesthetics of an adolescent’s varicocele and for these concerns to not be dismissed.

Beyond filling gaps in factual knowledge, many individuals used social media to find shared experiences among a common community, allowing them to communicate with their peers, seek advice, and receive emotional support. In this way, peer sexual communication provides a wealth of experiential information that could help adolescents manage anxiety and cope with uncertainty [25]. Prior studies have shown that young people frequently go online to ask if their sexual situations are “normal” or socially acceptable [10]. Advice from “similar others”—those with a common experience, background, and views—is more powerful than advice from experts when it comes to online health information [25,26]. Results from our analysis may inform strategies for enhanced communication with adolescent patients with varicocele.

Our study is not without limitations. First, Reddit is an anonymous forum on which information is self-reported and therefore not standardized, leaving much individual patient data unavailable. However, anonymity also allows for unfiltered discussions that many patients would feel uncomfortable with in a professional, in-person setting. Next, there are several steps where sampling bias may have occurred. Discussion forums likely overrepresent adolescents with higher-grade varicoceles, who may be more likely to undergo procedural management. Lastly, while MEM/PCA provides an objective method to analyze textual data, it may be limited in rendering textual valence and context in many of the posts. However, classic qualitative thematic analysis filled in that gap in offering insight. Future work is underway to use more advanced NLP models to provide nuanced contextual understanding, which may help bridge the gap between quantitative and qualitative analysis.

### Conclusions

This work sheds light on themes of discussion among male adolescents with varicoceles, an under-studied population. Though online discussion boards offer adolescents an anonymous environment to address sexual health issues, available information may be inaccurate. As a result, adolescents may receive misleading information, be given incorrect diagnosis or treatment recommendations, and may be steered toward unstudied supplements. Considering how frequently adolescents engage with social media, it is important to understand their use of networking sites so that physicians are better equipped to address adolescent concerns surrounding sensitive topics such as varicoceles, are prepared to dispel myths discovered online, and can improve communication and interactions with this patient population. Informed by these findings, a potential avenue for future research is to use a qualitative approach to understand perspectives of adolescents undergoing urology evaluation for varicoceles.

## Appendix

Multimedia Appendix 1 Posts that met inclusion criteria have been included as a supplementary file. To uphold privacy, we have made access restricted.

## Abbreviations

KMO: Kaiser-Meyer-Olkin
MEH: Meaning Extraction Helper
MEM: meaning extraction method
NLP: natural language processing
PCA: principal component analysis

## Data Availability

The datasets generated during and/or analyzed during this study are available from the corresponding author on reasonable request.
